# Pandemic Leadership: Is It Just a Matter of Good and Bad?

**DOI:** 10.1007/s11115-023-00712-6

**Published:** 2023-04-22

**Authors:** Titik Setyaningsih, Indra Bastian, Choirunnisa Arifa, Fuad Rakhman

**Affiliations:** 1 Faculty of Economics and Business, Universitas Gadjah Mada, Sosio Humaniora Street Number.1, Bulaksumur, Depok, Sleman, Yogyakarta, 55281 Indonesia; 2 Vocational School, Universitas Sebelas Maret, Kolonel Sutarto Number 150, Surakarta, 57126 Indonesia

**Keywords:** Pandemic, Governmental, Leadership, Coronavirus

## Abstract

This study aims to explore governmental leadership in response to global pandemic. A total of 52 articles from Scopus database were analyzed using Leximancer 4.51, followed by a content analysis. The findings are: (1) There are two concept changes, namely crisis labeling and a change in the meaning of masculine versus feminine which is embedded in leader’s decisions; (2) The theories that support the phenomenon of leadership are quite diverse; (3) The most common methodology is case study. The limitations of the study, especially in the articles analyzed during the coronavirus outbreak. We suggest future research directions into historical perspective.

## Introduction

Since the 2019 coronavirus (COVID-19) first struck the global community and was subsequently proclaimed a pandemic by the World Health Organization (WHO) in January 2020, every country has attempted to reduce the rate of transmission. Some countries have managed to stop the virus from spreading, while others have failed. Although every country has its own distinct cultural, social, economic, and political circumstances, healthcare systems around the world are relatively similar in nature and performance. Healthcare reform debates are dominated by ideologically driven arguments, and the policies that follow lack focus on common performance and incentive issues. The same concerns about how health is financed, how funds are spent, how systems are structured and then reformed keep cropping up, albeit with different emphasis (Goddard & Bloor, 2018).

Unified budgets for medical doctors, community health services, and hospitals, with the flexibility to allocate resources based on local needs and circumstances, establish a collective responsibility for the spectrum of services offered, including access, quality, and outcomes. The most appealing opportunity is to improve the quality of healthcare for the community. Quality improvement is now a collective responsibility rather than an individual choice (Wilkin, 2002). However, there is always a gap between expectations and capability; this gap exists in normal circumstances and becomes even more obvious in times of crisis.

Many studies have been conducted to fill this gap, including comparative studies as a reflection of health systems between countries by (Aldridge & Sundarapandiyan, 1995; Okma et al., 2010; Burau, 2012; Saltman, 2012; Seeberg et al., 2014; Bump, 2015; Davidson et al., 2016; Feachem et al., 2017; and Thakkar & Sullivan, 2017). Some of the information that is interesting to evaluate is concerned with distortion and who makes decisions. This is therefore linked to leadership, particularly effective leadership to overcome the pandemic.

Leadership is important and intriguing to study further; the decisions made by a leader when an epidemic threatens the safety of people’s lives have different consequences. When lives are at stake, a good leader will provide a positive contribution for an organization or a country while a bad leader will cause a fatal outcome (Wilson, 2020). Several studies on leadership during a pandemic include Kleinhuber and Hermann (2020), Dirani et al. (2020), Hatcher (2020), Cherneski (2020), Chuang et al. (2020), Escotet (2020), Fitzgerald and Wong (2020), Grint (2020), Lee (2020), Jamieson (2020), Henrickson (2020), Siuda-Ambroziak and Bahia (2020), Thomson (2020), Vroman and Danko (2020), Spector (2020), Wilson (2020). This raises the question: Is it simply a matter of good and bad leaders (?), or is there more to it? Wilson (2020) himself contends that the decisions a leader makes and how they affect a country is still an ongoing topic of investigation. Furthermore, different approaches might offer different models, findings, and implications, and as such, the concept of successful leadership is still open to inquiry. The research questions of this study are: (1) what are the themes of pandemic leadership in handling COVID-19 in terms of concepts, theories, and methods? and (2) what are the future research avenues into pandemic leadership?

One example of previous research on authentic leadership in the health sector is a study by Malila et al. (2018), which employs a literature review as the method for analyzing quantitative articles and makes recommendations for further research employing the literature review method to analyze qualitative or mixed-method articles. The present study uses a literature review, as in prior studies, but attempts to map leadership during the pandemic without restricting the methodology used. The research questions answered are the research themes, which become the research topic, theory, methodology, and probability of concept shifting, and the discussion of future research.

## Methodology

This research uses a literature study which involved collecting articles published since the start of the COVID-19 outbreak. In order to depict leadership during the pandemic, the method employed was based on a previous study by (Tian & Huber, 2019) which combined bibliometric analysis with content analysis. The article collection focused on papers published in ‘Scopus’. Scopus is a search engine for scientific articles which many reputable international journals have joined. It provides an impact factor and separates search times, thereby creating very clear cut off time frames. The articles selected were limited to those that were published between March and November 2020.

**Fig. 1:**
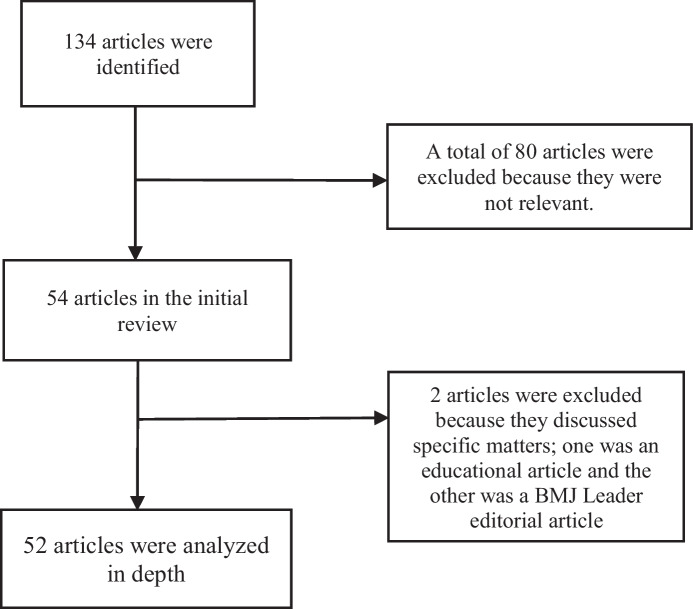
Literature review process

A total of 134 articles with the keywords "pandemic leadership" and "pandemic and leaders" were found. A total of 80 articles were excluded because they were not relevant. Two other articles were also excluded because they discussed specific matters; one was an educational article and the other was a BMJ Leader editorial article. The data were analyzed using Leximancer 4.51 software which produced a concept score and ranked the selected concepts. The analysis was carried out after adding the categories of "favorable" and "unfavorable". Subsequently, a content analysis was performed for in-depth identification. The following Fig. 1 is an illustration of the literature review process:

## Result

A total of 52 articles were downloaded as a result of the procedure described above, and a systematic analysis was conducted to determine the aim and main theme of each article. The distribution of articles from a selection of journals is shown below (Table 1):

**Table 1:** Article distribution

Journal	Number of articles
*American Review of Public Administration*	4
*BMJ Leader*	3
*Leadership*	3
*Human Resource Development International*	3
*Democratic Theory*	2
*Policy and Society*	2
*Politics and Gender*	2
*Sustainability*	2
*Gender, Work and Organization*	2
*Journal of Aging & Social Policy*	2
*Academic Medicine*	1
*AEM Education and Training*	1
*AJOB Empirical Bioethics*	1
*Cambridge Quarterly of Healthcare Ethics*	1
*Economic and Labour Relations Review*	1
*European Societies*	1
*European Journal of Sustainable Development*	1
*Gruppe Interaktion Organisation*	1
*Health and Sociology Review*	1
*International Organization*	1
*International Affairs*	1
*International Journal of Language and Communication Disorders*	1
*International Journal of Latin American Religions*	1
*Management and Organization Review*	1
*MAI Journal*	1
*Nonprofit and Voluntary Sector Quarterly*	1
*Paediatric Respiratory Reviews*	1
*Political Quarterly*	1
*Public Management Review*	1
*Prospects*	1
*Journal of Leadership Studies*	1
*Journal of Public Affairs Education*	1
*Journal of Educational Administration*	1
*Journal of Science Communication*	1
*Journal of Risk Research*	1
*Journal of Chinese Political Science*	1
*The International Journal of Community and Social Development*	1
**Total**	**52**

Source: Processed secondary data

**Fig. 2:**
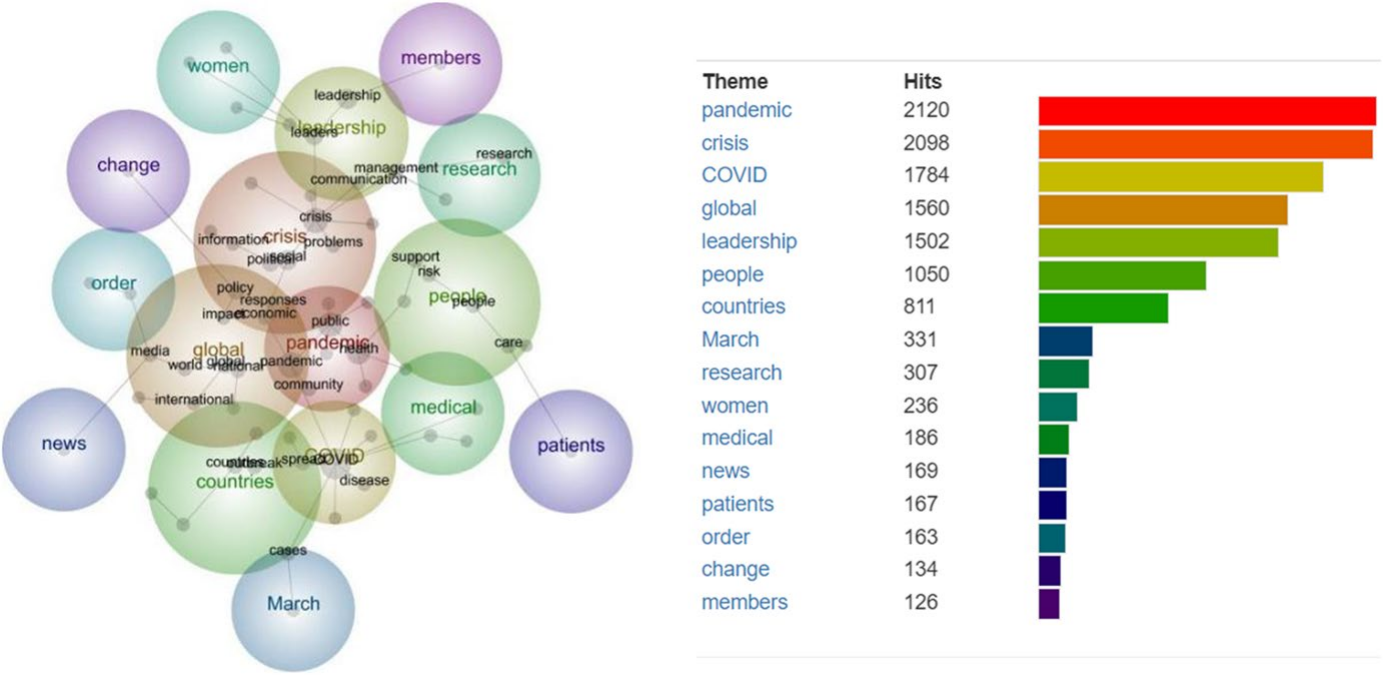
Concept analysis synopsis

According to the 52 articles, it can be seen that the analyzed themes are *pandemic* (2120 hits), *crisis* (2098 hits), COVID (1784 hits), *global* (1560 hits), *leadership* (1502 hits), *people* (1050 hits), *countries* (811 hits), *March* (331 hits), *research* (307 hits), *women* (236 hits), *medical* (186 hits), *news* (169 hits), *patients* (167 hits), *order* (163 hits), *change* (134 hits) and *members* (126 hits). There are approximately 3904 hits when the pandemic and COVID themes are combined. In addition to being a global research trend, the theme of leadership is an interesting topic to study at this time and is ranked fifth among the top five topics to study (Fig. 2).

In terms of ranked concept, the top five positions are held by COVID (1527), March (331 or 22%), China (189 or 12%), United Kingdom, or the UK (164 or 11%), and Trump (154 or 10%). The Word-Like Concepts are pandemic (930 or 61%), favorable (918 or 60%), unfavorable (859 or 56%), crisis (849 or 56%), public (804 or 53%), leadership (750 or 49%), health (698 or 46%), social (570 or 37%), and people (382 or 25%). China is the third most studied issue because the virus's initial propagation began in Wuhan and later grew to become a worldwide pandemic. America (when governed by former President Donald Trump) and the United Kingdom are the countries with the highest mortality tolls.

**Fig. 3:**
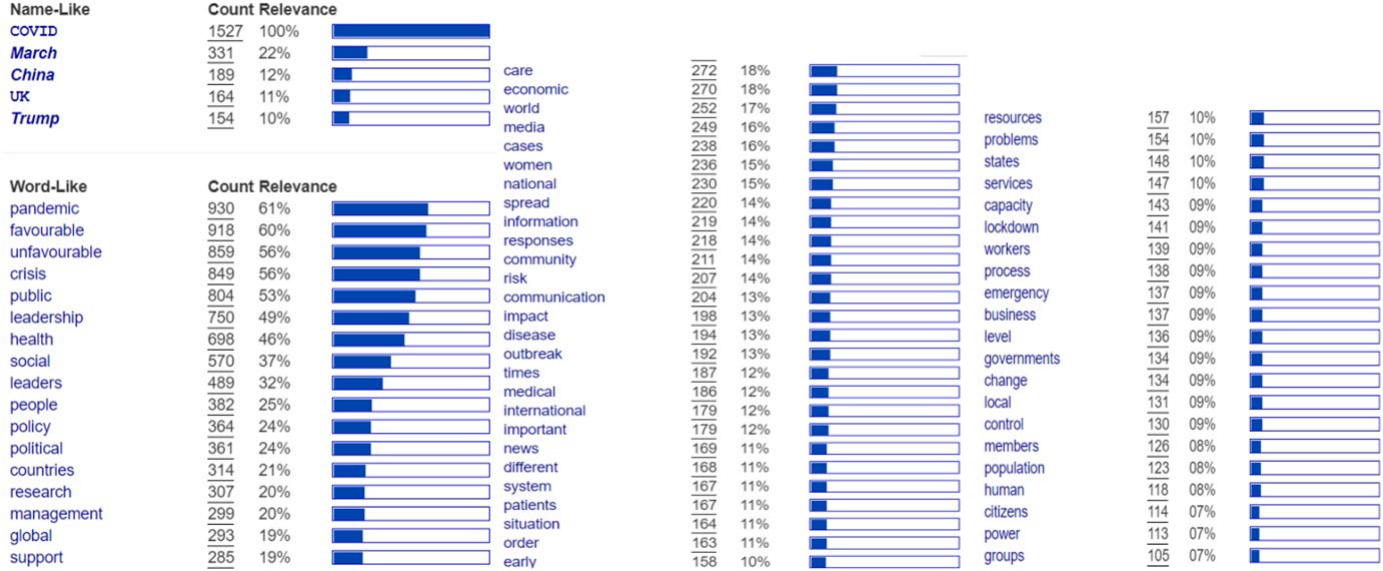
Ranked concept

Other themes that were discussed include: *policy, politics, countries, research, management, global, support, care, economic, world, media, cases, women, national, spread, information, responses, community, risk, communication, impact, disease, outbreak, times, medical, international, important, news, different, system, patients, situation, order, early, resources, problems, states,* and *services*. Themes that rank below ten percent are *capacity, lockdown, workers, process, emergency, business, level, governments, change, local, control, members, population, human, citizens, power* and *groups* (Fig. 3)*.*

**Fig. 4:**
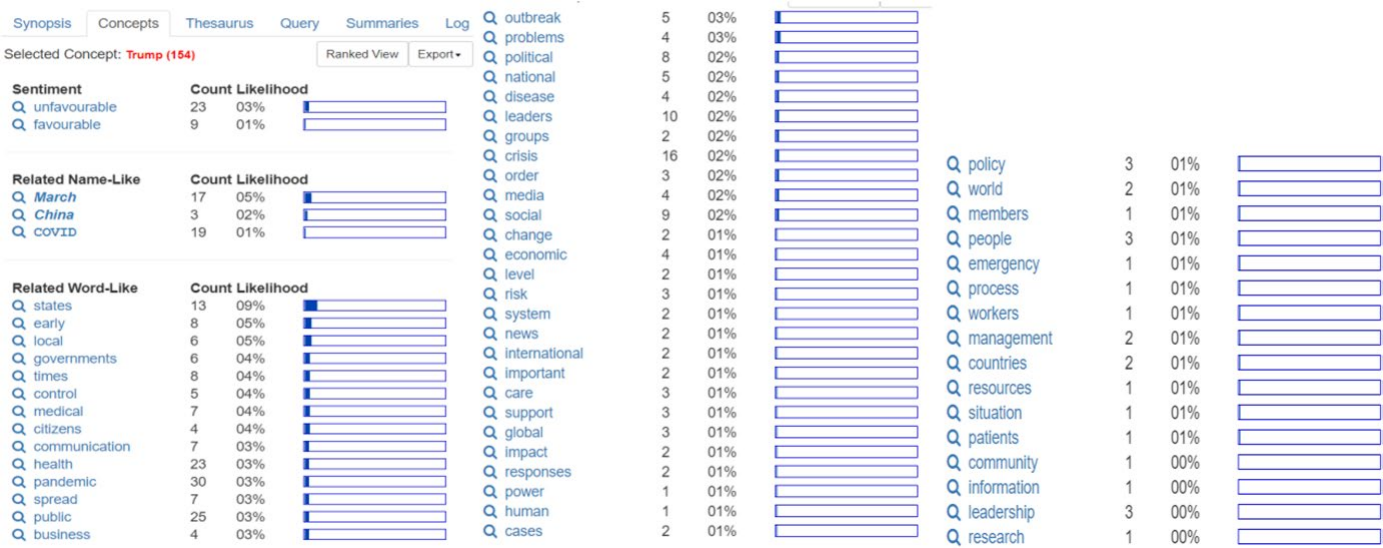
Trump

Interestingly, when the “favorable” and “unfavorable” analyzes of former United States President Donald Trump were added, the analysis revealed that Trump is considered unfavorable (23 counts), compared with 9 counts for favorable reports. Related name-like concepts include March 2020 (March) which has 17 counts, and COVID with 19 counts. Related word-like concepts include states (13 counts), health (23 counts), pandemic (30 counts), public (25 counts), leaders (10 counts), crisis (16 counts), communication (7 counts) and leadership, (3 counts or 0%) (Fig. 4).

**Fig. 5:**
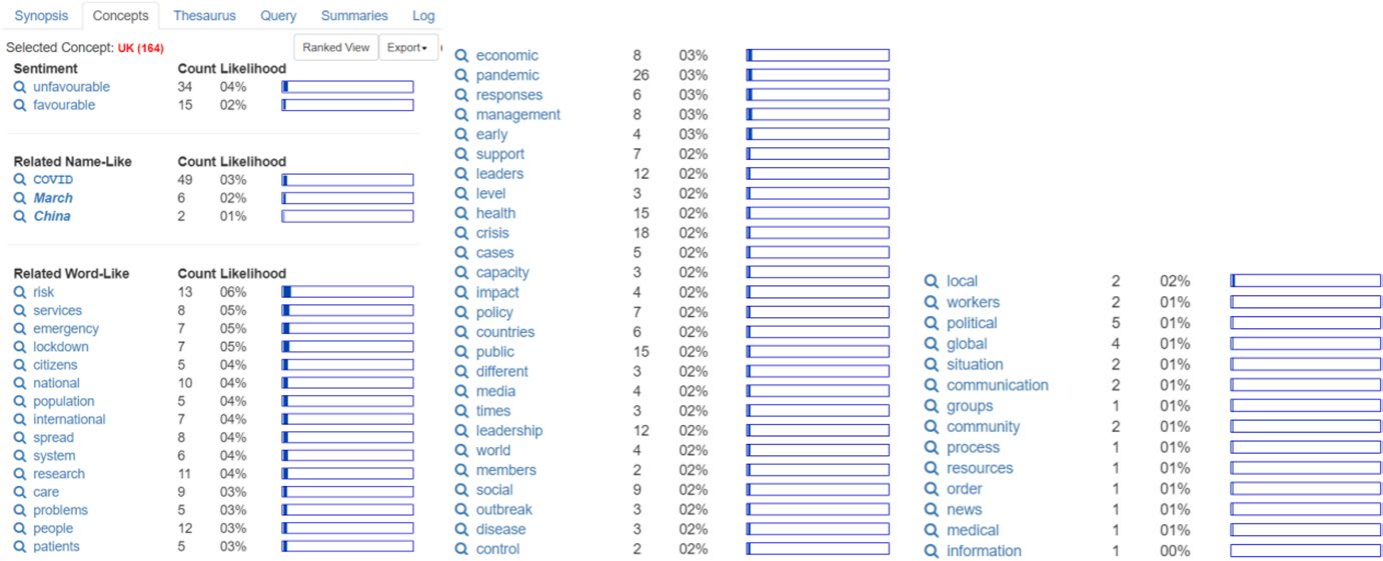
United Kingdom

A similar result was found for the UK. The unfavorable sentiment shows 34 counts, compared with 15 counts for favorable sentiment. Related word-like concepts include 13 counts for risk, 12 counts for people, 15 counts for health, 18 counts for crisis, 12 counts for leadership, and only 2 counts each for communication and community (Fig. 5)*.*

**Fig. 6:**
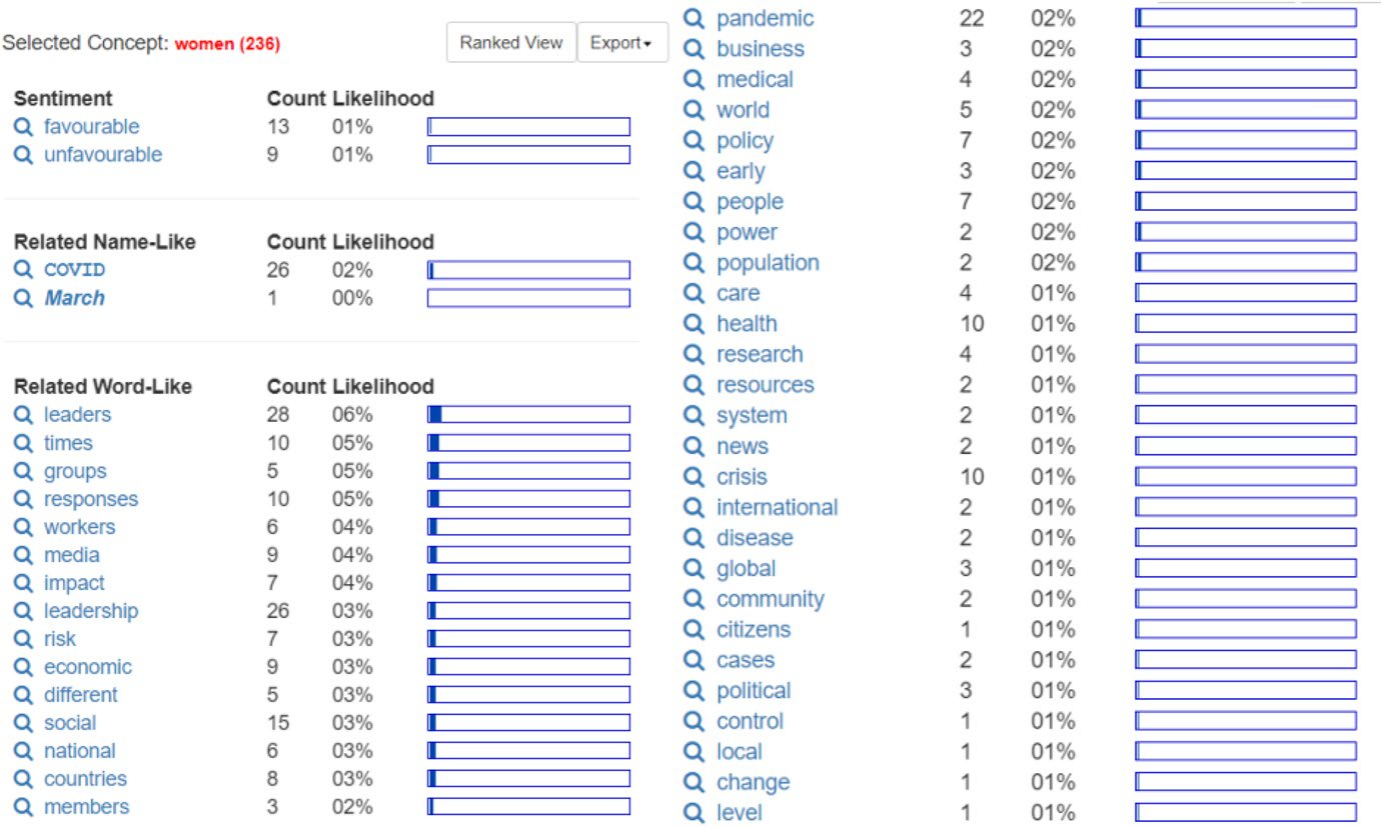
Women

**Fig. 7:**
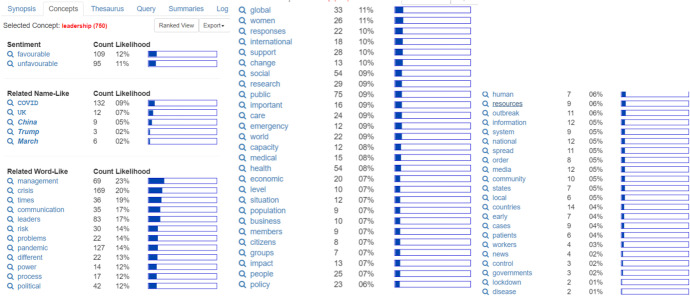
Leadership

The female theme, which received 236 hits, yielded some interesting findings. Leadership appears to be viewed through the lens of gender. Female leadership is claimed to be "favorable" (13 counts) rather than "unfavorable" (9 counts), even though it was ranked tenth in the previous concept synopsis. Female leadership appears to be remarkable during the pandemic, as seen from the related name-like concept COVID which has 26 counts. When this is combined with the word-like concept pandemic (with 22 counts), the result is 48 counts. In the related word-like concepts, leaders play a prominent role in mobilizing citizens (28 counts). If this is combined with leadership (26 counts), it results in 54 counts for the theme of women. This theme is related to times, responses, and crises (each with 10 counts), and how they affect the social sector (15 counts), health (10 counts), economic, and media (each with 9 counts). Details can be seen in Fig. 6 below:

The leadership theme ranks fifth in the concept ranking synopsis (1502 hits), with 750 hits in selected topics. Some of the most widely studied concepts use the words leadership, leaders, management, crisis, pandemic, communication, social, and health. Leadership is said to be “favorable” (109 counts), rather than “unfavorable” (only 95 counts). The topic of how leaders handle a crisis (pandemic) is still being investigated. The word "management" is a related word-like concept that appears frequently (69 counts). At the same time, in response to the research topic that should be given more emphasis, the social theme (54 counts) is given the same amount of attention as health (Fig. 7).

**Fig. 8:**
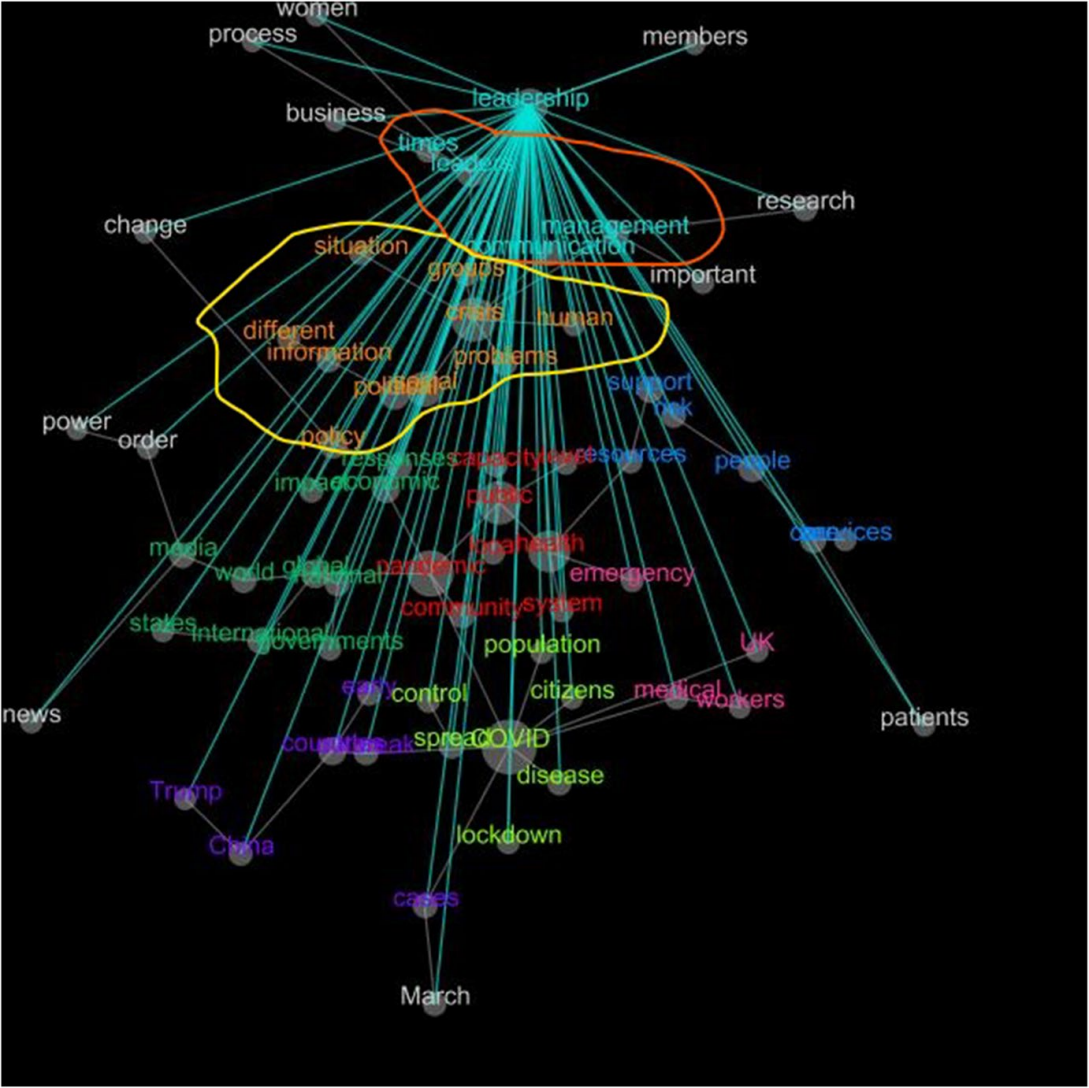
Leadership—conceptual map

The words leaders, leadership, times, management, and communication are all addressed in the conceptual map on the theme of leadership. This viewpoint argues that leaders must find strategies to manage resources and communicate with the community while considering the consequences of proper decision making (concepts are indicated by the area bounded by red pen). Furthermore, leadership (the concept is indicated by the area bounded by yellow pen) will also consider the situation, crisis, groups, safety of citizens (humans); face problems that require immediate solutions, different approaches; manage information submitted; make relevant policies which are very likely to include political elements. Process, business, women, members, research, importance, change, power, and order are some of the topics that are related to leadership with words (in white) (Fig. 8).

## Content Analysis

A pandemic is a global catastrophe, and the leaders of every country must continuously endeavor to take the necessary precautions to ensure the safety of their citizens. The theme of COVID-19 has sparked a lot of research in recent years, with China, the United Kingdom (UK), and the United States being among the most affected countries. As of December 21, 2020, the United States continues to lead the world in coronavirus mortality, with 317,684 deaths and 17,847,629 cases, greatly outnumbering New Zealand, which has 25 deaths and 2,121 cases (see https://coronavirus.jhu.edu/).

### Good and Bad Leadership

Transformative leadership to build collective (public) trust was developed into a good practice pandemic leadership framework (Wilson, 2020) which analyzes Jacinda Ardern's leadership in New Zealand. Key leadership practices consist of the government's readiness to be led by experts, as well as the government's attempts to mobilize the population and promote community resilience in managing their lives. All these practices are used to earn the trust required in leadership for transformative and collective action, such as the demands that arise during the pandemic. New Zealand appears to be on track to accomplish its goal of rapid and comprehensive control of the COVID-19 pandemic, rather than simply flattening the curve as other struggling countries have done (Wilson, 2020). The framework offered provides a perspective for other countries to study.

Meanwhile, (Wardman, 2020) proposes thirteen good leadership strategies: (*planning and preparing, narrating a clear-sighted strategy, meaning-making, direction giving, differentiating people’s needs, credibility and trustworthiness, transparency, openness, partnership and coordination, empathy, solidarity, being responsive and adaptive, media engagement across traditional and digital platforms*) to give leaders in the UK advice on how to strengthen understanding of decision-making, community resilience, and crisis-adjustment capacity. Effective communication also helps to develop good leadership, as it builds public trust via openness and transparency (Haÿry, 2020). The five best leadership practices that can be applied during normal times are modelling the way, inspiring a shared vision, challenging the process, enabling others to act, and encouraging the heart. During the pandemic, leadership practices have undergone adjustments, which include becoming a sense maker, technology enabler, promoting emotional stability and employee well-being, developing innovative communication, and maintaining the financial health of an organization (Dirani et al., 2020)*.*

Resident leadership or hospital leadership is preferred for hospitals by building emotional intelligence in the medical team (Rabin et al., 2020; Ward, 2020). In a medical team, emotional intelligence has two dimensions: awareness (self and social awareness) and regulation (self-management and relationship management).

In Latin America, the health crisis is the result of a long-standing loss of democracy, as well as economic pressure and political crisis. At first, especially in countries where democracy is already eroding, leaders may be tempted to take advantage of the crisis and use instruments such as emergencies to remove obstacles to their governance. Because of the nature of Latin American presidential institutions, the incumbents’ behavior can pose a threat to democracy when they are both too strong and too weak. Overall, the initial months after the COVID-19 outbreak show that populist attempts to use the pandemic issue as a mobilization strategy may backfire, whilst a more pragmatic response results in higher public approval. While the pandemic prevents protests, it has the potential to exacerbate political polarization and crisis. No matter what emergency solution is employed, the economic crisis is visible on all fronts. When a government fails to deliver effective solutions to social and economic challenges, people's faith in democracy may erode further (Weiffen, 2020).

### Change of Concept

The labeling of "crisis" during a pandemic is described as a subjective claim because there is influence and power for the occurrence of the claim (Spector, 2020). The current catastrophe is caused not just by a pandemic, but also by a moral crisis (Escotet, 2020). There is a paradox of globalism and nationalism that occurs simultaneously which is reflected when reporting a global pandemic, for example in China. Existing literature demonstrates that audience design influences and determines the target audience's choice of concepts and words. For example, accusations from some Western countries and the demonization of China and its politics have exacerbated China's growing displeasure with the current international system. China's objective to ensure its expanding power is reflected in official media portrayals of China as a defender of multilateralism and global governance, as well as a cooperative and responsible international stakeholder. This demonstrates China's inferiority in the right of international political debate when fighting the global pandemic (Yang & Chen, 2020).

Defining masculinity versus femininity, Jacinda Ardern's leadership is more masculine because it is more scientifically and ethically accountable (Henrickson, 2020; Jamieson, 2020; Wilson, 2020). Angela Merkel's leadership (Germany) assured the availability of intensive care facilities, and Taiwanese President Tsai Ing-wen's leadership (Taiwan) succeeded in strengthening public hygiene measures. (Cherneski, 2020). New Zealand, Germany, and Taiwan are examples of a leader's success in overcoming a pandemic with lower population mortality than the United States, the United Kingdom, and Italy. Women's communal leadership style produces benefits such as emotional responses that meet growing organizational needs, adapting to significant disruptions, aligning individual perspectives with expectations, and controlling societal boundaries in the pursuit of leadership and organizational success (Vroman & Danko, 2020). The use of feminine and weak codes to label former US president Donald Trump and Justin Trudeau implies rational decision-making that fails to take place at vital periods due to a lack of information (Thomson, 2020). Even though Italy has seen a large number of deaths as a result of the pandemic, there are good aspects to the leadership in this country. For instance, in a case study that raises Parmon's Ethical Value Framework, specifically ethical leadership, integrity, social concern, tradition, wisdom knowledge, work creativity, life nature, health, and humanity are all mentioned (Centorrino, 2020).

### Theory Used

Some of the theories used by researchers are *social identity theory* and *motivational language theory* (Wilson, 2020), *role congruity theory* (Vroman & Danko, 2020), *market theory and compensational theory* (Siuda-Ambroziak & Bahia, 2020), *conspiracy theory* (Patel et al., 2020), *hegemonic stability theory* (Norrlöf, 2020), *contingency theory* (McMullin & Raggo, 2020), *grounded theory* (Tham et al., 2020), *stakeholder theory* (Centorrino, 2020) and *leadership theory* (Grint, 2020). Studies about the correlation of pandemic leadership and gender (women) are analyzed based on *sense-making theory and feminist theory* (Cherneski, 2020).

### Methodology Used

The methodology used is mostly case studies, including (Angel & Mudrazija, 2020; Ansell et al., 2020; Barnard, 2020; Barton et al., 2020; Behrens & Naylor, 2020; Bowling et al., 2020; Capano et al., 2020; Funk, 2020; Goniewicz et al., 2020; Guy, 2020; Hartley & Jarvis, 2020; Henrickson, 2020; Jamieson, 2020; McGuire et al., 2020; Patel et al., 2020; Thomson, 2020; van Barneveld et al., 2020; Weiffen, 2020; Wilson, 2020). Comparative case studies in the Asia Pacific region have also been conducted (Fitzgerald & Wong, 2020). Some researchers use the historical approach methodology (Ikegbu et al., 2020; Shay, 2020; Vroman & Danko, 2020; Yang & Chen, 2020) while a foucauldian study was conducted by (Gjerde, 2020). The use of heuristics was applied by (Wardman, 2020) to study leadership in the UK.

Another researcher using a literature review method but within the scope of a country is (Patel et al., 2020), who conducted a study in Ukraine that found reports of disinformation during the pandemic. Qualitative studies were conducted by interviewing physician leaders in hospitals in Singapore (Tham et al., 2020), England (Patterson et al., 2020), New York (Chuang et al., 2020), and Canada (Cherneski, 2020).

## Discussion

 Some leaders are reported to have failed to overcome the pandemic, such as former US President Donald Trump, whose refusal to listen to expert advice caused many people to die from the coronavirus (Hatcher, 2020; Rutledge, 2020). Boris Johnson (UK) even focused solely on capitalism, profit generation, and privilege (Lee, 2020), in complete contradiction to the policies pursued by the New Zealand government. Low public trust can also trigger ineffective leadership (Hartley & Jarvis, 2020). The Prime Minister of New Zealand, Jacinda Ardern, is said to be a leader who managed to overcome the pandemic by building public trust (Wilson, 2020). However, Jacinda Ardern's policies must be modified for Maori in the long term (Kukutai et al., 2020).

Society interprets a leader's decisions and policies differently. During a pandemic, women must take the lead. (Rubenstein et al., 2020). Leaders must endeavor to plan ahead of time for a pandemic and any potential crises that may arise in the future (Mazey & Richardson, 2020). Leaders are no longer classified based on their gender, but rather on their actions.

It is important to anticipate community disinformation so that negative consequences do not worsen the burden on citizens, particularly those who are most vulnerable in conflict areas. Leaders must also be willing to collaborate without being constrained by bureaucracies (Johnson, 2020; Rehill et al., 2020). There are two communication recommendations, namely enhancing transparency by verifying health crisis messages and resolving leadership gaps that can be trusted to offer regional information on resources and support during the pandemic (Patel et al., 2020).

Social and health problems are two major issues that must be resolved first, and the impact of the policies implemented by the government will have a snowball effect that hits the economy of a country. Leadership in a smaller setting, such as a hospital, entails the tasks of meaning-making, sense-making, and decision-making, all of which are accompanied by a learning process (Kleinhuber & Hermann, 2020; Tham et al., 2020). As a result, while certain theories, concepts, and methodologies may be ideal for specific countries and cultures, they may produce different outcomes when applied to different cultures and conflict-prone countries. The impact of leaders' decisions is still being researched, not only in the short term (during the pandemic) but also in the long term (after the pandemic has ended), along with possible preventative measures if another crisis occurs in the future. Even though the term leadership is not new, this study adds to the leadership literature from a pandemic viewpoint.

## Conclusion

Pandemic leadership is not only about who is a good leader and who is not. Changes in the notion of gender (masculine versus feminine) and the labeling of crises are no longer analyzed straightforwardly. Whoever leads must be able to manage the people's public trust. Because the theories that support it are so numerous, the leadership phenomenon must be studied from a variety of perspectives. Case studies are the most common methodology employed, but global leadership challenges will allow for comparative case studies.

This study has limitations in that it only looks at articles from a specific period, specifically those connected to the COVID-19 pandemic (March to November 2020) so that it has not been linked historically to pandemic events. Future research could add to the analysis by tracing the pandemic's history, for example from the 1918 influenza pandemic to the present. Aside from the identified limitations, this study is not linked to other notions such as educational leadership. According to (Fernandez & Shaw, 2020), academic leaders who use a style of servant leadership can emphasize empowerment, involvement, and collaboration so that they can use emotional intelligence to put the needs of others ahead of their own. Furthermore, academic leaders should assign leadership responsibilities to team networks across the company to increase the quality of crisis resolution decisions and to communicate effectively and regularly with all stakeholders via multiple communication channels (including, in this case, country leaders who oversee pandemic leadership). Both content analyses are highly likely to contribute to surviving and passing the critical period. Finally, while this study focuses on the leader's perspective, which may differ from the follower's perspective (in the sense of the individuals being led), future research might focus on the follower's perspective or a combination of both.

## Data Availability

All articles included in this study are accessible through the Scopus database.
